# Correlates of vaccine protection against *Mycobacterium avium* sub-species *paratuberculosis* infection revealed in a transcriptomic study of responses in Gudair^®^ vaccinated sheep

**DOI:** 10.3389/fvets.2022.1004237

**Published:** 2022-11-24

**Authors:** Auriol C. Purdie, Karren M. Plain, Hannah Pooley, Douglas J. Begg, Kumudika de Silva, Richard J. Whittington

**Affiliations:** Sydney School of Veterinary Science, The University of Sydney, Sydney, NSW, Australia

**Keywords:** Johne's disease, *Mycobacterium avium subsp. paratuberculosis*, sheep, transcriptomics, Gudair^®^ vaccine, correlates of protection, vaccination, pathways analysis

## Abstract

A critical hindrance in the development of effective vaccine strategies to combat infectious disease is lack of knowledge about correlates of protection and of the host responses necessary for successful adaptive immunity. Often vaccine formulations are developed by stepwise experimentation, with incomplete investigation of the fundamental mechanisms of protection. Gudair^®^ is a commercially available vaccine registered for use in sheep and goats for controlling spread of *Mycobacterium avium* sub-species *paratuberculosis* (MAP) infections and reduces mortality by up to 90%. Here, using an experimental infection model in sheep, we have utilized a transcriptomics approach to identify white blood cell gene expression changes in vaccinated, MAP-exposed Merino sheep with a protective response in comparison to those vaccinated animals that failed to develop immunity to MAP infection. This methodology facilitated an overview of gene-associated functional pathway adaptations using an *in-silico* analysis approach. We identified a group of genes that were activated in the vaccine-protected animals and confirmed stability of expression in samples obtained from naturally exposed commercially maintained sheep. We propose these genes as correlates of vaccine induced protection.

## Highlights

- Vaccination with Gudair^®^ reduces paratuberculosis mortality but doesn't eliminate transmission.
- Within a flock some sheep will continue to shed *Mycobacterium avium* sub-species *paratuberculosis*.
- We identify gene expression differences between sheep with protective vs. unprotected responses.
- We propose use of identified genes as correlates of vaccine protection.

## Introduction

*Mycobacterium avium* sub-species *paratuberculosis* (MAP) infections in ruminants, particularly cattle, sheep, and goats, remain a source of increased mortality and reduced production in the global livestock industry (1–4). MAP is the causative agent of the disease known as paratuberculosis (Johne's disease, JD) which is an enteritis with granulomatous features. Infection follows oral exposure from colostrum or feces (5), but in severely affected cows, transuterine infection of the fetus can occur (6). Paratuberculosis in sheep has a long subclinical or latent stage of months to years (7) that is often accompanied by intermittent shedding of infectious MAP in the feces. Clinical disease manifests as wasting and is eventually fatal (8). Accurate, early diagnosis of subclinically infected livestock is not possible with current technology, leading to difficulties in disease control through test and cull approaches at population level, and creating a role for vaccination.

Following exposure, MAP bacilli transition to the host's gut mucosa where they interact with M cells and antigen-presenting cells and traffic to local lymphoid tissues, from which the intracellular mycobacteria are spread in blood and the lymphatics to other tissues (9). Mycobacterial interaction with dendritic and macrophage cells leads to activation of an immune cascade; primarily the initiation of *T* cell responses [reviewed in (10–12)]. As in many diseases, there is incomplete understanding of immune protection, particularly of the reasons for progression (or not) from early-stage silent infections to fulminant disease. While the early stages of JD do include cell-mediated immune responses through T helper 1 (Th1) cytokines (13) and the late clinical stages include antibody mediated responses, there is considerable animal to animal variation (14). Immune regulation begins early after MAP exposure of sheep and includes MAP-specific stimulation of peripheral blood mononuclear cells (PBMC) (15), interferon gamma (IFNγ) (14) and interleukin 10 (IL-10) (16). Retrospective analysis of data collected from both exposed and control sheep between 2 and 12 months post-exposure to MAP over a number of trials led to the finding that those that became infectious and progressed to clinical disease had early lower IFNγ responses than other sheep (17). In contrast, some animals successfully limited shedding of MAP from an early stage, reflected in low concentrations of MAP DNA in their feces, and this was accompanied by greater interleukin-10 production. The overall findings suggest that significant immune mechanism associated genomic changes occur during the pathogenesis of paratuberculosis, even at a very early stage of the disease process and although paratuberculosis is primarily a mucosal site associated disease, these changes may be detected in the host blood (17, 18).

Vaccination is commonly used in Australia to control MAP infection in sheep and goats. The commercial vaccine Gudair^®^ is widely used for their management and at present is the only vaccine approved for paratuberculosis in sheep and goats in Australia and New Zealand. The Gudair^®^ vaccine comprises of an inactivated (killed) Mycobacterium paratuberculosis strain 316 F combined with a mineral oil adjuvant that may be administered as a single subcutaneous 1 ml dose in lambs from 4 weeks of age and before 16 weeks of age (19).

Gudair^®^ substantially reduces mortalities Some farms have also been able to eliminate Mptb shedding or at least reduce shedding to below detectable levels, but animals from other Gudair^®^ vaccinated flocks continue to shed Mptb organisms in their feces and remain infectious for several years (20–23). The reasons for these differences in vaccine effectiveness between flocks remain unclear, however, differences in management, biosecurity practices, host genome or pathogens infecting these flocks may contribute to these differences.

Research conducted in the past 15 years since the commencement of vaccination in Australia has shown that vaccination with Gudair^®^ reduce mortalities attributable to MAP infection by up to 90% (23, 24). Whilst some farms have been able to eliminate or reduce MAP shedding to below detectable levels, vaccinated sheep on other farms are shown to continue to shed MAP in their feces and remain infectious (20–22, 24). Longitudinal studies have demonstrated that regardless of consistent use of vaccination as a strategy to control disease, some farms struggle to eliminate disease (23). The explanation for differences in vaccine effectiveness between flocks remains unclear, however, variances in management, biosecurity practices, host genome or concomitant pathogens infecting these flocks may contribute and in experimental trials, animals that succumb to disease despite vaccination present measurable variations to immune responses within a few months of pathogen exposure (25).

Vaccine formulations are often developed by stepwise experimentation, with incomplete investigation of the fundamental mechanisms of protection. Many effective licensed vaccines induce both cellular and humoral immunity as well as immune memory (26). However, studies aimed at obtaining a more detailed understanding of the mechanisms by which vaccine formulations enhance and support both innate and adaptive host immune defenses could identify correlates of protection potentially leading to the design of more effective vaccines. Targeted concentration on a limited selection of immune system components is useful for understanding specific interactions in the process of vaccine mediated protection but may not provide insight to correlates of protection. This requires a systems immunogenetics approach to investigate host- pathogen interactions, an approach recently taken to explore correlates of immunity in relation to hepatitis C virus (27). Gene interaction networks derived from transcriptomic gene expression analysis illustrate a logical scaffold for understanding cell biology, and these data, when analyzed in combination with peripheral (blood) measured immune parameters may identify key host responses and reveal interesting opportunities for targeted vaccine research.

We have previously reported significant CD4+, B, and *T*-cell proliferative responses in sheep vaccinated with Gudair^®^ that were not protected against infection (25). These studies were based on an experimental infection model in sheep that permitted accurate assessment of disease outcome. This was aligned with immune parameters such as lymphocyte proliferation, serum antibody and cytokine production levels at an early time point, 13 weeks post exposure to MAP (25).

The aim of this study was to investigate the transcriptome associated with protective immunity to MAP infection following Gudair^®^ vaccination, where efficacy was assessed through using the experimental infection model in Merino sheep and measuring gene expression from blood derived white blood cell RNA to encapsulate the host immune responses. The expression of selected genes of interest have been validated in samples obtained from both the experimental MAP challenged sheep and naturally exposed commercially maintained sheep. We have identified key pathways and genes regulated in response to successful vaccination.

## Methods

All animal procedures were approved by the Animal Ethics Committee, University of Sydney (2017/1245).

### Ovine Johne's disease model

#### Experimental trial

The study design has been previously described in detail (25). Briefly, a controlled experimental model of ovine JD was conducted utilizing a validated sheep infection model (28). Merino lambs were obtained from a commercial farm determined to be free from MAP infection [OJD Market Assurance Program Monitored Negative 3 (MN3), indicating three negative tests of flock by pooled fecal culture or serology over 4 years (29)].

Selected lambs between 2 and 4 months of age were transported to a JD-free quarantine farm at the University of Sydney, Camden, NSW (Australia) managed under conventional Australian sheep farming conditions. The lambs were separated into groups using systematic random sampling and administered a single 1 ml dose of Gudair^®^ (Lot No. B7846-08006, Zoetis, Australia) at the base of the neck. Lambs in the MAP-exposed groups were exposed to a low passage laboratory seed stock culture of MAP sheep (S) strain (Telford 9.2) at 0-, 1-, and 4-weeks post-vaccination, in total three oral doses of the MAP inoculum (total 2.74 x 10^9^) were administered over the period of 31 days. This trial was replicated 1 year later and samples for the transcriptomic study and the subsequent qPCR validation were sourced from both trials.

#### Farm study

To assess host responses to vaccination under natural conditions and to inform on-farm management of ovine paratuberculosis, four commercial enterprises with a practice of routine vaccination of lambs (between 4 and 16 weeks) with a single dose (1 ml) of Gudair^®^ in rural Victoria (Australia) were selected for a single sampling event (blood and feces).

Farms were selected if they met the following criteria: (a) farmers were willing to participate in the study, (b) flocks had at least one positive OJD diagnosis, (c) flocks had sufficient numbers of sheep to sample (*n* ≥ 350) and (d) flocks had been reported by the farmer as being consistently vaccinated with Gudair^®^ for at least 5 years. Thirty sheep between 2 and 4 years of age were selected and sampled per farm (Table 1). Samples sourced from the properties were utilized to test (qPCR), the use of the previously selected genes as correlates of vaccine efficacy in commercially managed properties.

**Table 1:** Prevalence of Johne's disease on-farm.

**Farm site ID**	**Prevalence in test cohort**	**Disease classification**	
		**HTJ positive**	**HTJ negative**
D	10%	3	27
E	3%	1	29
F	93%	28	2
G	20%	6	24

### Sampling

#### Experimental trial

Blood, serum, and fecal samples were collected prior to inoculation with MAP then repeated every 1–3 months post inoculation (PI) until necropsy to monitor the progress of the infection as described (30). At the conclusion of the trial at 49 weeks post MAP exposure (sheep aged 12–17 months), the sheep were necropsied under controlled conditions. Feces, blood and tissue samples were collected (12 tissue samples were collected from each animal at the terminal ileum, anterior ileum to posterior jejunum, mid distal jejunum, middle jejunum, mid proximal jejunum and anterior jejunum, and associated lymph nodes plus a section from three sites not associated with the intestines; the hepatic and prescapular lymph nodes, and liver). Viable MAP in feces was detected by radiometric BACTEC culture and growth confirmed by IS900 PCR (31) and quantification of MAP DNA was assessed by IS900 qPCR high throughput direct (HTJ) assay (32, 33). All collected tissue sections were cultured by the radiometric BACTEC method (34) and duplicate sections were fixed in formalin, sectioned at 5 μm, stained with haematoxylin and eosin or the Ziehl–Neelsen stain and graded following Perez et al. (35, 36). Disease status was defined by a histopathology lesion score where multibacillary (score 3b), or paucibacillary (score 1, 2, 3a, 3a-b, 3a-c, and 3d) was based on published criteria (36) with modifications to account for predominantly submucosal lesions (score 3d), transitional lesions (3a-b, 3a-c) and non-specific focal granulomatous lesions containing debris (score 0.5), as well as the number of acid fast bacilli observed.

#### Farm study

Fecal and blood samples were collected from 30 Gudair^®^ vaccinated sheep (age 2–4 years) on each of the four commercial enterprises at a single time point. Blood samples were collected into PAXgene^®^ tubes which are optimized for stabilization of intracellular RNA for subsequent isolation from whole blood for RT-PCR and stored at−20°C. MAP DNA was extracted from fecal matter for the IS900 qPCR HTJ assay quantification (32, 33).

### Classification of disease spectrum

#### Experimental trial

Merino sheep vaccinated with Gudair^®^ and exposed to MAP post-vaccination were monitored to determine their disease outcomes utilizing established classification metrics (35) including evidence of a positive tissue culture result for MAP at the cessation of the trials. Animals were classified as infected if they returned a positive MAP culture result for the intestinal tissue samples and/or terminal ileum and anterior jejunum lymph node samples sourced from necropsied animals (37), 26% (*n* = 9) of the exposed and Gudair^®^ vaccinated sheep were classified as infected and 74% (*n* = 26) as non-infected.

The infected group of sheep manifested a diverse range of disease severities; three had histopathological lesions consistent with either multibacillary (*n* = 1, lesion grade 3b) or severe paucibacillary (*n* = 2, lesion grade 3c) disease (8) and the remaining six had lesion grades of ≤ 2 for two or more intestinal tissue samples. Six were positive for MAP DNA in the feces by qRT-PCR detectable MAP growth (HTJ assay, average positive DNA quantity >0.001 pg). None of the nine animals selected for transcriptomic analysis within the non-infected group manifested any sign of infection; all animals within the non-infected group (*n* = 26) were fecal culture negative at all timepoints tested and had no JD histopathological lesions evident in the intestinal tissues.

#### Farm study

Prevalence of disease in the commercially raised sheep was determined by the presence of MAP DNA in the feces (Table 1).

### Study design for gene expression profiling

The samples studied by transcriptomic analysis (microarray) and real time quantitative PCR (qPCR) were sourced from animals at 13 weeks post MAP exposure, based upon findings from our previously reported study (25). Eighteen of the trial animals were selected for transcriptomic analysis from the vaccinated/MAP exposed cohort; nine were classed as infected therefore an equivalent number of uninfected animals were also selected (35).

### Preparation of white blood cells (WBC)

White blood cells (WBC) were isolated from peripheral blood samples utilizing a previously described method (38). Briefly, 9 ml blood was collected into EDTA coated blood vacuette tubes by jugular venipuncture and the buffy coats were isolated from each sample by centrifugation (1,455 x*g* for 20 min at 22°C). Residual red blood cells were lysed using ammonium chloride (AMCO) lysis following which WBC were pelleted by centrifugation (233 x*g* for 10 min at room temperature) and stored at−80°C until required for RNA preparation.

### Preparation of RNA

#### Experimental trial

Total RNA was isolated from WBC samples utilizing the proscribed method for the RNAspin Mini RNA isolation kit (Illustra, GE Healthcare). Quantification and integrity of the RNA samples was determined by NanoDrop 1,000 spectrophotometry (ThermoFisher Scientific) and Agilent 2001 Bioanalyser analysis with an acceptable RIN number between 6.0 and 10.

#### Farm study

RNA extraction was performed using the PAXgene^®^ Blood RNA kit, according to manufacturer's instructions. The resulting RNA was verified by NanoDrop 1,000 Spectrophotometer to obtain an RNA concentration.

All RNA samples were stored at−80°C prior to use. Where possible, all transcriptomic processing was conducted at the same time to prevent risk of machine/handler error.

### Microarray procedures

Affymetrix^®^ Bovine GeneChip^®^ 3'IVT arrays were used to analyse transcriptomic variations in experimentally vaccinated and MAP exposed Australian Merino sheep and controls. The Bovine Genome Array consists of 23,000 gene transcripts and includes approximately 19,000 UniGene clusters. Transcriptomic analysis was carried out at the Ramaciotti Center for Genomics (UNSW Sydney, Australia).

At the time the RNA samples were hybridized to the array GeneChips^®^, the Bovine array was determined to contain the most comprehensive coverage and a decision was made to match array and sample processing methodology with that of a concurrent project (18) to minimize variability and allow for cross analysis between studies. In the intervening time, Affymetrix developed an Ovine GeneChip^®^ Gene 1.0 ST Expression Array and further there has been development of RNA Seq technologies that allow for sequencing of the whole transcriptome however, capacity within Affymetrix Netaffx Analysis Center for Bovine Affymetrix^®^ GeneChip^®^ 3′IVT array probe set matches to both the Ovine GeneChip^®^ Gene 1.0 ST Expression Array and any mammalian orthologs thus ensuring all bioinformatic analysis was performed on correctly identified sheep relevant accession identities and probe sequences.

### Gene expression data analysis

The Affymetrix GeneChip^®^ operating software (GCOS) derived raw expression values (.CEL files) were analyzed by Partek Genomic Suite 6.6 software (Partek Inc.) using a bioinformatic strategy previously described in detail (18). Briefly, raw data was normalized using the RMA (Robust Multichip Averaging) algorithm (39) and correlation between data derived from separate arrays was confirmed by Principal Component Analysis (PCA). Lists of genes/probe sets significantly different between non-infected vaccinated sheep that were protected from disease in comparison to the infected vaccinated group were generated through testing analysis of variance (ANOVA) with a nominal alpha value set to 0.05 and Benjamini and Hochberg Multiple testing correction to reduce the false positive rate (40).

Gene identity annotation was performed based on similarity scores in Basic Local Alignment Search Tool (BLASTN) (National Library of Medicine, United States) comparisons against ovine or bovine sequences in GenBank. Gene lists containing significantly changed genes were submitted to GenBank for annotation based on similarity scores in BLASTN comparisons against bovine and ovine sequences and to Affymetrix Netaffx Analysis Center for Bovine Affymetrix^®^ GeneChip^®^ 3′IVT array probe set matches to both the Ovine GeneChip^®^ Gene 1.0 ST Expression Array and mammalian orthologs through searches of gene names, gene symbols, probe set ids, Gene Ontology (GO) and Medical Subject Heading (MeSH) terms, accession identities and probe sequences. These additional gene identifiers were incorporated into the subsequent functional analysis.

### Pathway analysis

Generated gene lists were analyzed for biological relevance through the use of IPA (QIAGEN Inc., https://www.qiagenbioinformatics.com/products/ingenuitypathway-analysis) utilizing techniques recommended by the program developers (41), The outline of the bioinformatic approach utilized is outlined in detail in our previous publication describing use of transcriptomics to assess adaptation of gene expression profiles during subclinical MAP infection in unvaccinated sheep (18) however the variables tested in the current study compare the RNA from a mixed cell population (WBCs) sourced from non-infected vaccinated sheep that were protected from disease to an infected vaccinated group.

### Real-time quantitative PCR (QPRC) analysis

qPCR analysis was performed to verify the array findings and to test the capacity of the selected genes as putative biomarkers of vaccine efficacy. RNA samples from all animals within the trials (*n* = 35) and those sourced from four commercially managed farms (*n* =120) were subjected to qPCR analysis. Three previously validated reference markers (18) were utilized following assessment by geNorm (Table 2); this follows MIQE (Minimum Information for Publication of Quantitative Real-Time PCR Experiments) guidelines (42).

**Table 2:** Primer sequences for qPCR validation.

	**Primer sequence (5'**−**3')**		
**Gene name**	**Clinical trial**	**Farm**	**Accession #**
LXN	F-taatagccgtctgccaaagg	F-agttcgacatttagcctgggt	NM_001080340
	R-ggcaccactgctgttgataa	R-agtcatcatttctttgcacttgct	
RARRES1.2	F-gcagtgtcaagcagtggaaaac	F-gcagtgtcaagcagtggaaaa	NM_001075430
	R-tactaagctccgtcagtgcc	R-accaagtgaatacggcaggg	
LYZ1.1	F-gatggcaaaacccctaacgc	F-gctgtagcatgtgcaaagcat	NM_180999
	R-agggagcaaccctccacata	R-ttacagggagcaaccctcca	
TNFRS21.2	F-ggacttgggaatctgctggaa	F-gcaatggccacggtattgac	NM_001076911
	R-ggacacaaacccaaacttgca	R-ccgtgtacccgttggagaaa	
TET2.1	F-gccacaccccagctttaga	F-ggctaaacagctgccagaactt	XM_001790146
	R-tacccttctgtccaaacctttct	R-cgaagagcctggataagggc	
HbF1.1	F-gagaacttcaggctcctggg	F-gagaacttcaggctcctggg	NM_001014902
	R-attggcaacaccagtcacca	R-gtgatatctgtgggccaggg	
TES.2	F-tattgtcacgtgtggcttcc	F-cacccagcttgtttcgtctg	Bt.26803.1.A1_at
	R-aatggaaaccgtgcaaaaag	R-atccttgacacaccacagcg	
C10H15orf48	F-gcttcgtcattcgctgtgta	F-tcgaaaaaccgatgtgatcctt	XM_004010648
	R-cggaccttctgcaactcttc	R-agaaaaaggcgaggactggt	
IP-10 (CXCL10)	F-gctactgacagtttcctccc	F-cccacgtgtcgagattattgcc	NM_001046551
	R-agaatatgggccccttggag	R-agctgatttggtgactggctt	
BOLA1a.1	F-attgggatcgaaacacgaga	F-tcaccctgagatgggaacct	NM_001038518
	R-ctctaccgtcgtagccgaac	R-tcctccagatcacagctcca	
**Reference genes**	
PPIA	F-tgagcactggagagaaaggatttg	F-tgagcactggagagaaaggatttg	NM_178320
	R-agtcaccaccctggcacataa	R-agtcaccaccctggcacataa	
Ovine B-Actin	F-catcctgaccctcaagtacc	F-catcctgaccctcaagtacc	
	R-ctgttgtagaaggtgtggtg	R-ctgttgtagaaggtgtggtg	
H3F3a	F-gaggtctctataccatggctc	F-gaggtctctataccatggctc	NM_001014389
	R-gtaccaggcctgtaacgatg	R-gtaccaggcctgtaacgatg	

#, number.

Ten genes of interest were selected for validation of the array data (Table 2). Forward and reverse primer pairs were designed for the gene regions of interest using the online software Primer 3 (43) and Primer-BLAST (https://www.ncbi.nlm.nih.gov/tools/primer-blast/). Where possible, primers were designed across an intron-exon boundary or included intronic sequences, to prevent amplification of genomic DNA and the alignment relevant to the *Ovis aries* region of interest was confirmed through use of Basic Local Alignment Search Tool (BLASTN) (National Library of Medicine, United States).

The technique utilized for qPCR assessment of the selected RNA samples is described in detail (18), briefly 5 μg of template RNA was reverse transcribed to cDNA using oligo(dt), random primers and the AffinityScript qPCR cDNA synthesis kit Stratagene, Agilent. qPCR was performed using an M x3,000 P Real-time PCR system (Stratagene, Agilent) using the QuantiTect SYBR Green PCR kit (Qiagen). Data was analyzed using qBASE + analysis software (Biogazelle) utilizing a modified Comparative Ct (ΔΔCt) method (44).

Testing of capacity of the selected genes as putative biomarkers of vaccine efficacy was performed on the samples sourced from four commercially managed farms (*n* = 120).

## Results

### Identification of genes associated to a protective profile

Transcriptomic analysis was performed on RNA isolated from WBC sourced from 18 Merino sheep sampled at 13 weeks post MAP exposure (classified infected *n* = 9, non-infected *n* = 9). Following statistical analysis of Affymetrix^®^ GeneChip^®^ derived data, genes meeting the criteria for differential expression (*FDR* ≤ 0.05 and a *FC* ≥1.4) were identified for the non-infected vaccinated sheep that were protected from disease in comparison to the infected vaccinated group (Table 3). The non-infected sheep returned 65 differentially regulated gene probes (23 downregulated and 43 upregulated) indicative of a correlate of vaccine protection in the case of MAP infection.

**Table 3:** Genes differentially expressed in the Non-Infected vaccinated sheep (protected) compared to the Infected vaccinated sheep.

**Gene symbol**	**Gene title**	**P value**	**Fold-change**
BOLA-DQA1	major histocompatibility complex, class II, DQ alpha, type 1	5.06E−03	−3.2
LYZ2	lysozyme C-2	5.07E−03	−2.8
RARRES1 (TIG1)	retinoic acid receptor responder 1 (tazarotene induced 1)	2.35E−02	−2.1
HMGB3	high mobility group box 3	2.10E−02	−2
HBG	hemoglobin fetal subunit beta-like	5.37E−02	−1.8
HbF1.1	hemoglobin, gamma	2.68E−02	−1.7
LXN	Latexin	4.39E−02	−1.6
LOC100850064	versican core protein-like	3.82E−02	−1.6
HBG	hemoglobin, gamma	4.33E−02	−1.6
C1QA	complement component 1, q subcomponent, A chain	2.75E−03	−1.6
TRB@	—	5.10E−03	−1.5
TNFRSF21	tumor necrosis factor receptor superfamily, member 21	5.10E−03	−1.5
TET2	tet methylcytosine dioxygenase 2	4.48E−02	−1.5
C4BPA	complement component 4 binding protein, alpha	4.48E−02	−1.5
NRP1	neuropilin 1	2.10E−02	−1.4
C10H15orf48	chromosome 10 open reading frame, human C15orf48	3.98E−02	−1.4
MRPL13	mitochondrial ribosomal protein L13	2.68E−02	−1.4
APP	amyloid beta (A4) precursor protein	3.86E−02	−1.4
SERPINI1	serpin peptidase inhibitor, clade I (neuroserpin), member 1	2.68E−02	−1.4
AQP11	aquaporin 11	7.51E−03	−1.4
PRPF40A	PRP40 pre-mRNA processing factor 40 homolog A (S. cerevisiae)	4.33E−02	1.4
SFRS18	splicing factor, arginine/serine-rich 18	1.62E−02	1.4
SLTM	SAFB-like, transcription modulator	8.98E−03	1.4
HELZ	helicase with zinc finger	5.37E−02	1.4
MPZL1	myelin protein zero-like 1	5.07E−03	1.4
LOC538749	disheveled-associated activator of morphogenesis 1-like	4.33E−02	1.4
DDX46	DEAD (Asp-Glu-Ala-Asp) box polypeptide 46	3.86E−02	1.4
CHD2	chromodomain helicase DNA binding protein 2	4.39E−02	1.4
S100B	S100 calcium binding protein B	2.68E−02	1.4
USP7	ubiquitin specific peptidase 7 (herpes virus-associated)	4.48E−02	1.4
ATRX	alpha thalassemia/mental retardation syndrome X-linked	3.98E−02	1.4
TBL1XR1	transducin (beta)-like 1 X-linked receptor 1	6.07E−04	1.4
ZNF292	zinc finger protein 292	5.07E−03	1.4
LOC532189	carboxypeptidase D-like	2.35E−02	1.5
RBM5	RNA binding motif protein 5	2.68E−02	1.5
SPATS2L	spermatogenesis associated, serine-rich 2-like	1.29E−02	1.5
BPTF	bromodomain PHD finger transcription factor	2.75E−03	1.5
ZNF644	zinc finger protein 644	2.35E−02	1.5
PPP2R2B	protein phosphatase 2, regulatory subunit B, beta	3.82E−02	1.5
RASA2	RAS p21 protein activator 2	7.51E−03	1.5
TCERG1	transcription elongation regulator 1	2.68E−02	1.6
SF3B1	splicing factor 3b, subunit 1, 155kDa	5.37E−02	1.6
MAPK6	mitogen-activated protein kinase 6	2.68E−02	1.6
SEC63	SEC63 homolog (S. cerevisiae)	1.62E−02	1.6
BOLA-A	major histocompatibility complex, class I, A	6.07E−04	1.6
TES	testis derived transcript (3 LIM domains)	6.07E−04	1.6
DPYSL2	dihydropyrimidinase-like 2	8.98E−03	1.6
AKR1C4	aldo-keto reductase family 1, member C4	3.71E−02	1.7
IP-10 (CXCL10)	interferon-gamma-inducible protein	4.39E−02	1.7
SAFB2	scaffold attachment factor B2	2.68E−02	1.7
EIF2AK2	eukaryotic translation initiation factor 2-alpha kinase 2	3.71E−02	1.7
PSMD8	proteasome (prosome, macropain) 26S subunit, non-ATPase, 8	3.82E−02	1.7
GBP4	guanylate binding protein 4	1.29E−02	1.7
MLL3	histone-lysine N-methyltransferase MLL3-like	2.75E−03	1.8
PAFAH1B2	platelet-activating factor acetylhydrolase 1b, catalytic subunit 2 (30kDa)	2.68E−02	1.8
RAD51AP1	rAD51 associated protein 1-like	2.68E−02	2.1
BOLA1a.1	MHC class I antigen clone 2	2.68E−02	2.2

These genes are indicative of a protective vaccine profile in the case of MAP infection.

The most strongly regulated genes were associated to the Major Histocompatibility complex (MHC) family. Genes within these domains contain individual proteins in which variable and constant regions encode conserved sequence patterns of immunoglobulin and immunoglobulin-like molecules (45, 46) well–reported as vital components of the immune environment and in particular with the antigen presentation process (47, 48). The mechanism of this process requires a sustained interaction with the antigen/MHC complex to generate proliferation and initiate downstream signaling responses (49).

Other notable genes modulated between the non-infected and infected vaccinated cohorts (Table 3) included IP-10 (CXCL10, FC 1.7), Retinoic acid receptor responder 1 (RARRES1/TIG1, FC−2.1), latexin (LXN, FC−1.6), hemoglobin genes (HbF1.1, FC−1.7 and HBG, FC−1.8), and TNF-receptor superfamily member 21 (TNFRSF21, FC-1.5). IP-10 presented enhanced expression in the non-infected compared to infected group, indicative of a role in the protective response. RARRES1 and latexin, members of the same gene family associated with cellular proliferation, were downregulated in non-infected sheep compared to the infected sheep, which could reflect decreased expression in those sheep protected from disease and/or enhanced expression in the infected sheep. Modulation of RARRES1 has been identified in response to IFN-γ production in other infectious diseases (50). TNFRSF21 and several hemoglobin genes including hemoglobin gamma, were also downregulated in non-infected sheep. These genes may be associated with lack of protection and/or disease susceptibility despite vaccination.

### Ontological analysis of differentially regulated genes

To gain an understanding of potential relationships between the differentially regulated genes, the list of significant genes from Table 3 were interrogated using the bioinformatics analysis tool IPA^®^.

The genes were probed based upon functional annotation and to assess if a functional gene category contained an over-representation of genes relative to the microarray reference gene list. This analysis identified cellular signaling (DPYSL2, IP-10/CXCL10, MPZL1, AKR1C3, RASA2, MAPK6, C1QA, NRP1, and APP) and cell death (TNFRSF21, SERPINI1, ATRX, PPP2R2B, TCERG1, NRP1, and APP) as the top functional gene ontology categories associated to vaccine-induced protection at 13 weeks post MAP exposure (Table 4).

**Table 4:** Gene Ontology functional categories for genes whose expression correlates to a protective profile of Gudair^®^ vaccination.

**GO categories**	**Function**	**P Value**	**Number of genes**
Cell-to-cell signaling and interaction	Communication/*T* cell migration	1.70E-02	9
Cell death and survival	Cell death/apoptosis	9.89E−03	7
Cell morphology	Branching	2.19E−03	5
Immune cell trafficking	Migration/cellular infiltration	9.33E−03	5
Cellular development	Differentiation	2.23E−03	5
Cell signaling	Homeostasis	9.66E−03	3
Cellular compromise	Disassembly	5.48E−03	3
Amino acid metabolism	Uptake	2.45E−03	2
Cell cycle	Mitosis	1.33E−02	2
Cellular assembly and organization	Accumulation	7.74E−03	2
Cellular movement	Chemotaxis	1.96E−03	2

**Figure 1:**
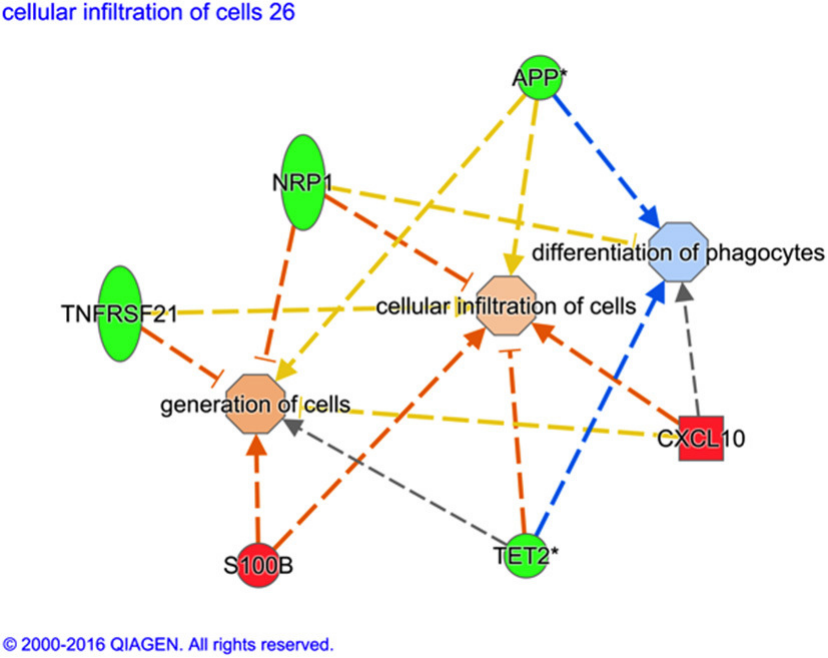
Variation of gene ontological (GO) functions associated to a protective vaccine profile. The cartoons illustrate expression changes in genes or functions associated to Non-Infected in comparison to Infected sheep. Genes and the predicted downstream response are highlighted in color. Color intensity indicates the degree of increased (red) or decreased (green) relative expression of the individual gene expression and predicted activation (orange) or suppression (blue) of downstream function as a consequence of the measured gene expression. Expression of the selected genes (S100B, TET2, CXCL10, TNFRSF21, NRP1, and APP) are predicted to activate generation of cells and cellular infiltration but suppress differentiation of phagocytes in vaccinated sheep that show no sign of MAP infection. *Indicates that multiple identifiers in the dataset file mapped to a sing gene identifier within the Global Molecular Network.

Although there is some overlap between the genes, the predicted downstream action of these gene combinations are not all the same, as illustrated in Figure 1.

**Figure 2:**
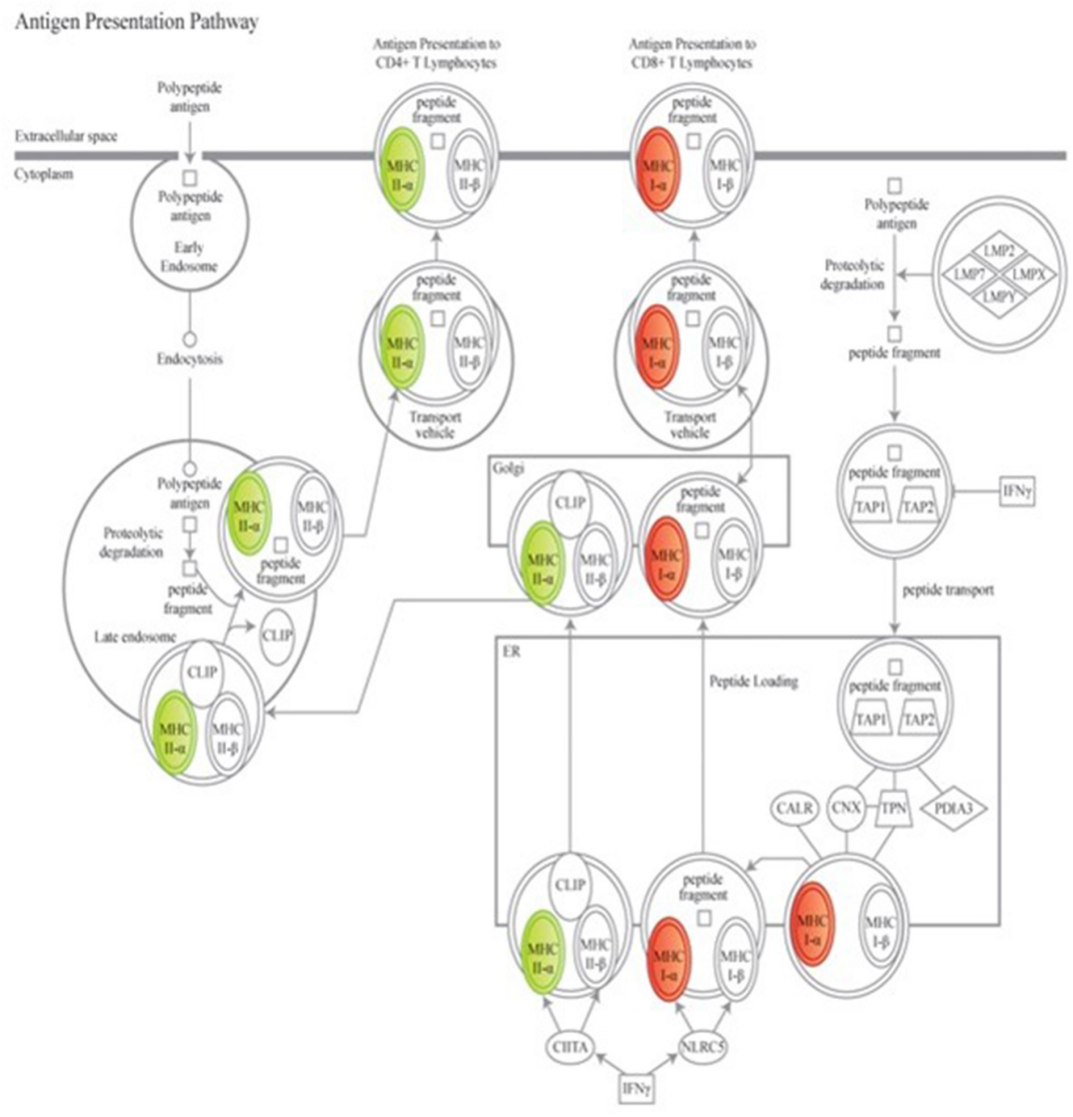
Genes associated to the antigen presenting canonical pathway are associated to a protective vaccine profile. The cartoons illustrate expression changes in genes associated to Non-Infected Gudair^®^ treated and MAP exposed sheep in comparison to similarly treated Infected sheep. Genes and the predicted downstream response are highlighted in color. Color intensity indicates the degree of increased (red) or decreased (green) relative expression and suggest a suppression of antigen presentation to CD4^+^ *T* cell lymphocytes but enhanced presentation to CD8^+^ *T* cell lymphocytes. Detailed explanation for figure notations is located at https://qiagen.my.salesforce-sites.com/KnowledgeBase/articles/Knowledge/Legend.

To further explore the biological meaning of the differentially expressed genes in this dataset, canonical molecular pathways associated to the experimental groups were analyzed using IPA. Based on prior findings reported by our group (38), of particular interest were the variations in overlaid gene expression results associated to the antigen presentation pathway (Figure 2). Data mining to determine downstream effects of the differentially regulated genes revealed that a protective vaccine profile involved a modified Pattern Recognition Receptor (PRR) canonical pathway and a modified Complement System canonical pathway.

### QPCR validation of selected genes of interest

Validation of transcriptomic/microarray predicted gene expression was tested and achieved using RNA samples sourced from all experimental trial animals (*n* = 35) at 13 weeks post MAP exposure (Table 5).

**Table 5:** Fold change comparison of the observed array gene expression and the qPCR validation of expression in experimentally exposed sheep.

**Gene name**	**Accession #**	**Microarray fold change**	**qRT-PCR fold change**	
			**Experimental trial**	**P value**
LXN	NM_001080340	−1.6	−1.5	0.038
RARRES1.2	NM_001075430	−2.1	−1.5	0.008
LYZ1.1	NM_180999	−2.8	−2.5	0.001
TNFRS21.2	NM_001076911	−1.5	1.2	0.231
TET2.1	XM_001790146	−1.5	−1.5	0.070
HbF1.1	NM_001014902	−1.7	−3.4	0.001
C10H15orf48	XM_004010648	−1.4	−1.4	0.001
TES.2	NM_001046390	1.6	1.2	0.750
IP-10 (CXCL10)	NM_001046551	1.7	2.1	0.030
BOLA1a.1	NM_001038518	2.2	1.2	0.164

#, number.

Use of selected genes as correlates of vaccine induced protection was tested in RNA samples obtained from sheep maintained under standard Australian commercial farming conditions (*n* = 120). The selected sheep had received Gudair^®^ vaccination at the recommended age (between 4 and 16 weeks of age) and were exposed to MAP through natural contact. Due to a paucity of HT-J positive animals in the individual farms (Table 1), statistical analysis of qPCR data was performed on the collated Farm Study data set rather than on the individual farms and compared non-infected vaccinated sheep (*n* = 82) with infected sheep (*n* = 38) cohorts (Table 6).

**Table 6:** Fold change comparison of the observed array gene expression and the qPCR validation of expression in a wider cohort of sheep (samples sourced from 4 commercially maintained farms).

**Gene name**	**Accession #**	**Microarray fold change**	**qRT-PCR Fold Change**	
			**All farms**	**P value**
LXN	NM_001080340	−1.6	−1.5	0.001
LYZ1.1	NM_180999	−2.8	−2.5	0.001
TNFRS21.2	NM_001076911	−1.5	−1.5	0.046
TET2.1	XM_001790146	−1.5	−1.6	0.001
C10H15orf48	XM_004010648	−1.4	−1.4	0.001
TES.2	NM_001046390	1.6	1.3	0.348
IP-10 (CXCL10)	NM_001046551	1.7	2.3	0.001

#, number.

## Discussion

The primary aim of this transcriptomics study was to exploit a unique resource in the form of blood derived WBC samples from Gudair^®^ vaccinated and MAP exposed Merino sheep with a view to identifying a gene expression profile consistent with a protective profile in response to vaccination. In addition, we sought to mine the data to gain a deeper understanding of the biological relevance of the modulated genes in the context of our previously reported study, in which we identified significant differences in the proliferation of CD4+, B, and *T*-cells over time in Gudair^®^ vaccinated sheep in which the vaccine failed to protect against infection compared to the non-infected vaccinated sheep (25). Pathway analysis seeks to associated differentially regulated genes with known ontological pathways and the conclusions drawn are based upon previously published literature that may have utilized variable cell populations. Any conclusions and assumptions of pathway associations utilizing a mixed cell population must acknowledge that further research may be required to determine the specific cell subsets from which differentially expressed genes derive.

Previous genomic studies suggest that in sheep and cattle, the early subclinical phase of Johne's disease pathogenesis is reportedly characterized by an increasing cell-mediated pro-inflammatory immune response largely driven by CD4+ T helper 1 (Th1) cytokines (13). In bovine Johne's disease evidence has been presented supporting a hypothesis that pathogenesis may be mediated predominantly by the loss of CD4^+^ T lymphocyte responses over the course of the disease (51). Our group has previously reported the results of a small scale transcriptomic study identifying subclinical gene expression in response to early MAP exposure in experimentally exposed juvenile Holstein cattle (38). The MAP exposed calves were selected with evidence of high IFNγ at 4 months post MAP exposure and of particular interest was the finding that following exposure to MAP, the host immune response appears to be driven to enhance the expression of genes related to the ability of cells to present antigenic peptides to CD8^+^ T lymphocytes rather than to the CD4^+^ pathway. We have since presented evidence that in sheep, this early subclinical high IFNγ profile is consistent with a reduced risk of disease susceptibility (25). Of great import, there is evidence from the present study that a protective vaccine profile mirrors the findings of the previously reported bovine transcriptomics study, supporting a role for a CD8^+^ dominant response in protection against MAP infection. CD8^+^ *T* cells are important for protective responses in tuberculosis, with the proposed mechanism involving cross-priming by dendritic cells *via* apoptotic vesicle uptake and MHC class I presentation (52). This also aligns with the identification of cell apoptotic pathways as one of the major cellular pathways affected.

In the present study, expression of MHC class I antigen (BoLA, FC +2.8) was enhanced in circulating WBC from non-infected compared to the infected sheep. The MHC class I protein product of this gene is a tissue antigen that mediates cellular immunity and represents a primary mechanism in the eradication of intracellular pathogens, *via* presentation of epitopes to the *T* cell receptors (TCR) of CD8^+^ *T* cells, a process that triggers programmed cell death by apoptosis. Conversely the most significantly downregulated (FC−3.2) gene in the non-infected group compared to the infected group was MHC class II, DQ alpha type 1 (BoLA-DQA1). The MHC II genes encode a protein that occurs as a chain composed of three domains (α1, α2, and α3) and typically occur on antigen presenting cells where it couples with epitopes of antigens (e.g., phagocytosed MAP). Epitope bound to MHC II is presented to naïve CD4+ T helper cells where it is recognized by the CD4 receptor and TCR, priming polarization of the *T* cell to either a memory or an effector T helper phenotype. Amongst other functions, MHC II mediates immunization/ immune tolerance to antigens and modulation of MHC class II gene expression may reflect changes in the cellular composition of the peripheral blood rather than decreased individual cell expression. An apparent gene expression-related suppression of CD4+ *T* cell responses through MHC class II downregulation is at odds with previous findings that showed enhanced proliferation of CD4+ *T* cell and *B* cells at 13 weeks post MAP exposure associated with a protective response in Gudair^®^ vaccinated sheep (25). However, deeper mining of the functions associated to differentially regulated genes predict both generation of T lymphocytes and the cellular infiltration of cells (Figure 1). This suggests that a protective vaccine profile at 13 weeks post MAP exposure involves enhanced accumulation of T lymphocytes and trafficking /migration of these cells.

A correlate of vaccine-mediated protection that involves enhanced cell accumulation is further supported in the transcriptomic evidence of activated cellular proliferation and pro-inflammatory cytokine associated immune responses to cells in the non-infected sheep. This pathway supports activation of NFκB; a transcription factor whose expression plays a critical role in the regulation of inflammatory processes (53). Binding of mycobacteria to macrophage surface receptors, especially TLR2 and TLR4, initiates a rapid pro-inflammatory response mediated by MAPK and transcription factors including NFκB (54). Previous studies have shown that NFκB activation leads to the synthesis of lysosomal enzymes, membrane-trafficking regulators and cytokines important for phagosome maturation and macrophage mediated killing of pathogenic mycobacterium such as *Mycobacterium avium* and *M. tuberculosis* (Mtb) (55, 56). Notably, IP-10 (C X CL10) expression was upregulated in non-infected vaccinated sheep, indicating an association with protective vaccine responses. IP-10 was first described for its chemoattractant properties and role in the recruitment of *T* cells to sites of inflammation, however IP-10 knock-out models have shown a broader involvement in effector *T* cell generation and CD8+ cytotoxic *T* cell function as well as cellular migration (57). IP-10 is an early IFN-gamma response gene that is enhanced in Th1-type inflammatory diseases and it has been identified as a potential biomarker of disease outcome in tuberculosis, able to differentiate between active and latent forms (58). Our findings suggest that IP-10 may be associated with cellular migration, CD8^+^ *T* cell and Th1 responses in vaccinated sheep and a potential biomarker of vaccine-mediated protection.

Hemoglobin genes were downregulated in the non-infected vaccinated group, which may reflect an alternate iron acquisition pathway utilized by mycobacteria to aid intracellular survival. Iron is essential to the survival of mycobacteria in the host however the transferrin/lactoferrin iron acquisition pathway requires high input energy by the mycobacteria. MAP has unique growth requirements *in vitro* related to iron acquisition, in that they require the addition of mycobactin, though possessing elements of the mycobacterial “iron box” (59). A separate heme pathway for iron acquisition has been identified in Mtb (60), with homologous genes found in MAP and other mycobacterial species; a secreted heme scavenger protein (Rv0203), transmembrane transporter proteins (MmpL11 and MmpL3) and heme degrading protein that releases bound iron (MAP0467c/MhuD). Acquisition of iron *via* this pathway is advantageous to the mycobacteria due to reduced energy input and increased iron availability, as the majority of iron in the host is associated with heme (61, 62). A number of other bacteria utilize this mechanism to acquire iron (63). Moreover, macrophages are involved in iron homeostasis *via* a variety of mechanisms, including turn-over of erythrocytes and hemoglobin-haptoglobin scavenging *via* the CD163 cell surface receptor (64, 65). The modulation of hemoglobin genes may reflect the utilization of host heme-associated iron in animals that develop disease, though it is not clear why these genes were related to fetal hemoglobin forms.

Of particular interest is the potential enhancement of complement mediated cellular opsonisation *via* genes associated with the classical pathway. Previous studies regarding the interaction of complement components with Mtb have recognized that the complement component C3 is an important mediating factor in macrophage phagocytosis of the mycobacterium (66). Mycobacterial entry into macrophages may be mediated by either non-specific pinocytosis (67) or *via* receptors (68) such as the complement receptor type 3 (CR3). CR3 mediated uptake involves cellular recognition of mycobacteria coated with the complement factor C3bi in an opsonic process also known as type II phagocytosis (69) in a mechanism that sees the mycobacteria sinking into the macrophage with the participation of the enzyme RhoA (a member of the Rho family of small guanosine triphosphatases) for the reorganization of filamentous actin structures. This process occurs without involvement of respiratory burst mechanisms or even co-stimulation with pro-inflammatory mediators (69–72). Several studies have suggested that opsonised Mtb preferentially utilize CR3 to enter the macrophage, although in the case of Mtb, CR3 deficiency does not significantly affect mycobacterial survival (66, 73). In this study we present evidence of differential activation of complement system associated genes as a factor in the vaccine associated protective profile, predictive of suppression of the C3 associated pathway, suggesting that a protective vaccine reduces the potential for mycobacterial opsonisation. There was decreased expression of complement component 4 binding protein alpha (C4BP, FC−1.5), a gene that encodes a multimeric protein controlling activation of the complement cascade through the classical pathway. In addition, there was decreased expression of complement component 1 Q alpha (C1QA, FC−1.6); a gene that encodes a major constituent of the complement subcomponent C1q. The downstream effects of these combined gene expression changes were associated to suppression of cellular phagocytosis and the immune response in cells as well as blocking the quantity of phagocytes. It is not known at this stage if the suppression of genes associated to the classical complement pathway translates to an effect on the traditional canonical pathway or if this modulation is an unknown pathway.

It must be acknowledged that in the intervening years since the inception of the initial experimental trial, transcriptomic technology has advanced and if this was to be repeated utilizing the Ovine GeneChip^®^ Gene 1.0 ST Expression Array or RNA-Sequencing, there may be variations in the findings however this multi-year project has resulted in previously unreported insights only made possible due to the interaction of multiple parties.

The results of these array experiments confirm and extend existing studies of MAP exposure and further, support the findings that we have previously presented in relation to a protective profile associated to successful Gudair^®^ vaccination. We present a group of genes whose expression is associated to a protective profile and are informative in elucidating which pathways are associated to a protective vaccine strategy. This knowledge may lead to improved vaccine formulations and development of diagnostic tools to identify poor vaccine responders whose removal from flocks may lead to reduction of sub-clinical disease and address the issue of disease persistence in vaccinated flocks.

## Data availability statement

The datasets presented in this study can be found in online repositories. The names of the repository/repositories and accession number(s) can be found in the article/supplementary material.

## Ethics statement

The animal study was reviewed and approved by University of Sydney Animal Ethics Board. Written informed consent was obtained from the owners for the participation of their animals in this study.

## Author contributions

AP, KS, KP, DB, and RW contributed to conception and design of the study. AP, KS, KP, DB, and HP assisted in collection of samples and associated studies to determine clinical status. AP carried out all transcriptomic based research and wrote the first draft of the manuscript. KP and AP carried out qPCR validation and statistical analysis. All authors contributed to manuscript revision, read, and approved the submitted version.

## Funding

This reported research was jointly funded by Meat and Livestock Australia, by the Cattle Council of Australia, the Sheepmeats Council of Australia, the Wool Producers Australia through Animal Health Australia, and by the University of Sydney under the following grants (P.PSH 0576 and P.PSH0813). Publication costs were supported by Sydney School of Veterinary Sciences.

## Conflict of interest

The authors declare that the research was conducted in the absence of any commercial or financial relationships that could be construed as a potential conflict of interest.

## Publisher's note

All claims expressed in this article are solely those of the authors and do not necessarily represent those of their affiliated organizations, or those of the publisher, the editors and the reviewers. Any product that may be evaluated in this article, or claim that may be made by its manufacturer, is not guaranteed or endorsed by the publisher.
